# Study on Situational Influences Perceived in Nursing Discipline on Health Promotion: A Qualitative Study

**DOI:** 10.1155/2013/218034

**Published:** 2013-09-02

**Authors:** Meimanat Hosseini, Tahereh Ashk Torab, Mohammad Hossein Taghdisi, Safar Ali Esmaeili Vardanjani

**Affiliations:** ^1^Department of Community Health Nursing, Faculty of Nursing and Midwifery, Shahid Beheshti University of Medical Sciences, International Branch of Shahid Beheshti University of Medical Sciences, Tehran, Iran; ^2^Department of Medical-Surgical, Faculty of Nursing and Midwifery, Shahid Beheshti University of Medical Sciences, Tehran, Iran; ^3^Department of Health Promotion, Faculty of Tehran University of Medical Sciences, Tehran, Iran; ^4^Shahrekord University of Medical Science, Shahrekord, Iran

## Abstract

*Introduction and Objectives*. Nurses, as behavioral models, play a key role in health promotion, and their attitudes towards health promotion highly influence their health and performance. The aim of this study is to explore nursing students' perception of studies in nursing discipline as a situational influence on health promotion. *Materials and Methods*. This study was conducted using directed content analysis, by means of 20 deep semistructured interviews with nursing students. The participants were selected on purposive sampling. Data was analyzed by the qualitative content analysis method. All interviews were recorded, transcribed, and reviewed, and all codes were extracted and summarized. The codes were subcategorized on the basis of centralization and were categorized after review of subcategories, and finally, a theme was determined. *Findings*. The theme of nursing discipline's situational influence on nursing students' health promotion was revealed. This theme consisted of “choosing the field,” “unfavorable environmental factors,” “negative impacts of studies in nursing discipline on health,” “positive effects of studies in nursing discipline on health”, “needs,” “attractiveness (aesthetics),” and “coping with negative situational influences in nursing discipline.” *Conclusion*. The perception of studies in nursing discipline as a health-promoting behavior is under influence of social environment. Considering the importance of the students' positive perception of the existing situation, it is essential to pay attention to their attitudes and perceptions so that they can provide better services to patients.

## 1. Introduction

The increased rate of health care costs and the concerns about inefficiency of patient care has led to greater support of health promotion concept and its propagation among health care personnel, particularly nurses [1].

Health promotion is a process which enables people to control their health [2]. Health-promoting behavior is an important concept in nursing [3] and should be considered as a healthy nursing or interactive method which promotes health [4].

Nurses are the biggest group to render services to patients [5] and take a significant role in public health promotion [6]. According to statistics published by Nursing Organization, nurses constitute 50% of the entire 600,000 personnel in Iran's health system [7]. Nurses, as behavioral models and professional caretakers, play a key role in health promotion, and their beliefs on the importance of health promotion are part of their work [8].

Pender states that cognitive variables such as situational influences along with particular behavioral variables can help determine health-promoting behaviors of people [9]. Situational influences directly [10] and indirectly affect the behaviors and the commitment to action planning [11]. These influences embrace health-promoting behaviors, conceptions of possible choices, need characteristics and aesthetic aspects of the environment [12]. Situational influences, such as choices, may increase or decrease commitment to or participation in health-promoting behaviors [13]. Since behavior is dependent on the surrounding environment, it may be affected by situational factors [14], and this influences a person's behavior [15].

 The results of studies have revealed that nursing students' view of occupational situation is highly personal. These students often think there is a relation between the status of a profession and judgments regarding the value of a job, and their attitude towards nursing is under influence of several factors such as negative attitude of parents and unreal images of nurses shown in TV [16].

In order to attract more people to nursing profession, a positive image of nursing needs to be developed by the public society and nursing education center. It should also be mentioned that experiences and concerns of nursing students are a permanent theme of professional pressure [17]. 

The considerable point is that according to studies, the conception of nursing affects the student's decision to enter, continue or discontinue nursing profession [18]. Unsupported by their families or their faculties, students have frequently made decision to continue or discontinue their studies in spite of their desires. A research on, 1,000 American students revealed that students believe that nursing includes a physical challenge and the respect for nursing profession is of a low level. On the other hand, another study has revealed that students have selected nursing discipline owing to good job opportunity, job safety, salary, and interest. Furthermore, friends, families, relatives, media, and society have been noted as contributing factors in choosing nursing discipline [17]. 

Nursing students should be able to do activity in cultural context of nursing profession easily and confidently. The students who feel accepted in this context experience less anxiety. On the other hand, immoral and inaccurate activities are connected with being not accepted in cultural groundwork [19]. 

In a broad library search, researchers found no research on the relation between study of nursing discipline as a situational factor and health-promoting behavior, neither in Iran nor in other countries. However, the shortage of nursing forces all over the world, including Iran, has been considered to be a serious challenge [20], the cause of which might be the effect of nurses' perception of nursing discipline on their health-promoting behaviors. On the other hand, it is not known what effect the perception of studies in nursing discipline has on students' health-promoting behaviors. Therefore, this research was conducted to explore nursing students' perception of studies in nursing discipline as a situational influence on health promotion. 

## 2. Materials and Methods

Nursing research, like nursing itself, deals with different and complicated phenomena [21]. The present study is part of a broader research which was performed by qualitative method with a content analysis approach. Qualitative content analysis is a story-like data content analysis in order to determine prominent themes and patterns among themes [22]. The phenomenon under study in this research is study of nursing discipline as a situational influence. 20 nursing students from nursing faculties of medical universities of the city of Tehran participated in individual semistructured interviews. The participants were chosen by purposive sampling method from June up to November 2012 until data saturation. After choosing the participants, interviews were performed in leisure times at classes or rooms allocated by managers of the faculties or work office of researcher or open area of the faculties, however the participants wished. In researches performed in natural environment, the main responsibility of the researcher is to obtain consent of the participants in a conscious way [22]. In line with this objective, after obtainment of verbal consent from participants and completion of letter of informed consent by them, deep semi-structured interviews consisting of four questions were performed based on the guidance of the interview's questions. Deep interview helps the researcher to make a mental interpretation from the stories told by participants and to assess the facts as they clarify the phenomenon under study. During the dialogue between interviewer and interviewee, meaningful and relevant subjects are produced [23]. Data was collected using deep, semi-structured interviews and making field notes. All interviews were recorded on an MP3 player. The interview's questions were based on health-promoting behaviors and delineation of nursing students' perception of their academic field as a situational influence on their health-promoting behavior. Minimum and maximum times of individual interviews were 19 : 30 and 71 : 25, respectively. To prevent data collection bias, all interviews were performed by one researcher. Among moral points of this research were compliance with the principle of secrecy and confidentiality of the participants' names and information in all steps, making them confident about protection of their secrets and the right of withdrawal in any part of the research. 

In this research, data was analyzed by directed content analysis method, in which primary encoding is started with a theory or relevant research findings [24]. The aim of directed content analysis is to validate or develop a conceptual framework or theory. The existing theory or research can help concentration on the research question. By using the existing theory or research, the researchers start with determined concepts and variables as primary coding classifications. Then, operational definitions of each category class are determined by using the theory [25]. Since Pender's model was the basis of this research on health-promoting behaviors, the researchers used directed content analysis, and categories were determined on the basis of Pender's Health Promotion Model [26].

Based on this, one of the researchers first performed an interview and made field notes and then studied them line by line accurately, underlining key words and sentences and coding each. After coding was completed, the interview was restudied to see if any possible code is found. Next, the codes were merged and summarized, and similar codes were classified. This way the primary classifications of the data were obtained. Then, the researcher tried to perform the same process for the categorizations as well. Finally, the subcategories were located in categories on the basis of common characteristics and dimensions. 

The rigor of qualitative research is shown through the researcher's attention to discovered information and its confirmation. The aim of accuracy in qualitative studies is to show the authenticity of participants' experiences. Credibility, dependability, confirmability and transferability are terms which constitute practical methods for supporting rigor of a research [27]. In this study, credibility of the data was achieved by long-term investigation of the research subject by researcher, allocation of sufficient time to gather data, interview, observation of the examinees during the sessions, data control by participants and review of extracted codes. To achieve dependability of the data, the researchers used the validity of research findings. To achieve confirmability of the data, audit trial was used. For this purpose, a detailed report of the process leading to conclusion has been presented, whereby enabling the pursuit of research process and actions taken by researchers. To achieve transferability of the data, data were accurately investigated by external supervisors.

## 3. Findings

Twenty nursing students from nursing faculties of medical universities of the city of Tehran participated in individual semi-structured interviews. The participants were 6 male and 14 female nursing students who were studying in the 1st to 8th semesters of Bachelor's Degree course of studies. Four of the participants were married and sixteen were single. Six of them were working as nurse, one was working in a profession other than nursing and thirteen were unemployed. Five participants were living in dormitory, fourteen were living with their families, and one was living with friends. 

Data analysis indicated that the main theme of nursing's situational influence on health promotion included 227 meaning units, 35 subcategories, and 7 categories as follows: “choosing the field,” “unfavorable environmental factors,” “negative impacts of studies in nursing discipline on health,” “positive effects of studies in nursing discipline on health,” “needs,” “attractiveness of nursing discipline” and “coping with negative situational influences in nursing discipline.”

Studying in university had been perceived as a situational influence on health promotion, in such a manner that one of the participants said that

*“Because of my age the only important thing is studying. Any other thing doesn't matter … educational future is important … that is why I say success in the most important aspect of life still makes me feel healthier.” *(Participant No. 10) **

The students said that they have used the subjects learnt in nursing discipline for their health promotion. Almost all participants in this research believed that studying in nursing discipline is a factor contributing to their health promotion. In this respect, a student said that

*“Well, the field we are studying is very effective in health promotion. It has helped me think more profoundly about the things which I used to skip very easily.” *(Participant No. 7) **

The use of subjects to diagnose disease and identify probable diseases by observing the patients hospitalized in hospital ward was mentioned as the health-promoting behavior.

*“Well, personally I try to review things learnt in the class by checking them in myself … anyway as we go to the wards and see the patients, it makes us think and see what problems we have and what problems we don't have*.” (Participant No. 2) **

The significant role of nursing education was a considerable aspect in paying attention to oneself and to family members and everyday activities in line with health promotion, which the participants believed in:

*“Well, the most important thing is the education I was given in all fields. Since I am married and have baby, I have to be more careful than others, in all my works … and the training I can give to my child, about what he should do.” *(Participant No. 8) **

### 3.1. Choosing Nursing Discipline

The conception of having a good occupational future and the feeling of safety owing to good financial situation were among the reasons for choosing nursing discipline, so that good job opportunity of this discipline had overcome the lack of interest in that:

*“You can be relaxed when you think no matter if you don't like nursing; there will be job for that … anyway you will have a job. I think one of the reasons; actually the main reason that I chose it was its good job opportunity”. *(Participant No. 13) **

Furthermore, there was a belief of having better situation in terms of health-promoting behaviors in the case of studying a better discipline:

*“If I hadn't come to university and hadn't had academic studies, I would certainly have had worse behavior. If I had studied a better discipline, my behavior would certainly have been even better and I would've had better mental conditions and better self-confidence.” *(Participant No. 1) **

### 3.2. Unfavorable Environmental Factors

The low status of nursing in public society had been perceived as a factor contributing to reduction of health level. The effect of lack of respect and value in public society was considered to be connected with field comparison:

*“When you go somewhere else like hospital or the public society, you see people treat nursing as a very ordinary job, not a job that is serving them, so they don't respect it.” *(Participant No. 1) **

Negative comments of medical personnel and the colleagues in regards to nursing were another issue that the participants considered as a negative impact on their health promotion. They believed that the biggest occupational damage is inflicted by their colleagues:

*“In hospital you see that both physicians and nurses have this opinion. I mean the nurses themselves inflict the biggest damage to this job. When you see such behavior you become very upset that even your colleagues don't respect you.” *(Participant No. 1) **

The negative attitude of the community, family, and relatives influenced the value of nursing discipline as stated by the students, the effect of which was shown by reduction of interest in nursing:

*“At first, I was very interested in nursing. But later when I saw the attitude of others, I started to dislike it. Now I don't like nursing at all. I mean the thing for which I made so much effort is now valueless for me.” *(Participant No. 10) **

### 3.3. The Negative Impacts of Studies in Nursing Discipline on Health

The students pointed to negative impacts of studies in nursing discipline, particularly in internship courses. These negative impacts were worse in internship courses in which they dealt with mental problems of patients:

*“Internship courses which deal with mental problems have a very bad psychological effect on nurses.” *(Participant No. 6) **

To see patients' pain and sorrow in clinical environments was mentioned as a factor influencing the students. This impact was conveyed to students through the feeling of sympathy and altruism:

*“Our work in hospital includes such sad experiences. For example, someone who had an accident came from another city to hospital in Tehran. He ordered one of his relatives in his city to buy platinum and bring for him in Tehran. But his relative was killed in his way to Tehran in an accident and his money was wasted as well. The poor guy experienced another disaster in addition to his accident. By seeing that, a feeling of sadness comes to your heart instinctively and unconsciously, because he is a human being like you.” *(Participant No. 5) **

The negative impacts of work in hospital were more severe in an internship course because, in addition to negative impacts of seeing patients and their problems on the students, it caused a feeling of depression and aimlessness:

*“The practical course we are passing has depressed us. In addition, we don't know exactly how we should pass this year.” *(Participant No. 3) **

Physical tiredness caused by intensive academic programs was another negative influence resulting from studies in nursing discipline, as the result of which the students could not do their daily affairs:

*“For example, during the course of this semester, when we came from university we were too tried. Sleeping was the only thing we could do. Well, no time remained for us to think about something or do something funny or think about our nutrition to see what our body needs.” *(Participant No. 14) **

The students who had emigrated from other cities to Tehran to study nursing expressed another problem as well. They said that limitations of living in dormitory and missing their family members influenced their health:

*“Living in dormitory has some limitations. Unconsciously, when a person is in some public place like dormitory, some works are limited automatically.” *(Participant No. 5) **

The negative impact of living in dormitory had been conceived as a shocking change which had made the participants unable to do any activity and had influenced all their life planning:

*“When I was in dormitory, I had free time but I didn't know who to do. I could neither do exercise nor study my lessons. At least in the first semester I was in such conditions. I was really in shock.” *(Participant No. 13) **

### 3.4. The Positive Effects of Studies in Nursing Discipline

The perception of being different from ordinary people of the society and having more knowledge was among positive effects of studies in nursing discipline. By way of better care of oneself, nursing discipline could have positive effect on health:

*“Well, I differ from other people. I have more knowledge on medical treatment. You imagine someone like my mother who has studied another field of study. Well, she has no much knowledge like me. She cannot take care of herself.” *(Participant No. 2) **

Performing examinations, familiarity with health standards, and preventive activities were among the positive things in nursing discipline. Awareness about health standards and activities resulted in compliance with health requirements:

*“I think the minimum difference between us and other people who don't have this information are that we know what things are good or bad for health; for example, we know what things cause problem for heart, kidney, etc. At least we know the standards, so we can comply with them.” *(Participant No. 6) **

The ability to diagnose diseases by help of academic studies in nursing discipline was another point which had positive effect on health promotion:

*“As a person who has studied nursing, I can easily diagnose diseases.”* (Participant No. 2) **

The arrival to nursing profession was a factor which increased the responsibility of the participants to both themselves and their family members. They felt that since they have chosen nursing discipline they will try more to promote their health level. The source of this effort was the insight in health-promoting behaviors:

*“Since I have studied nursing discipline and entered this profession, I am responsible towards my family as well as myself … before I entered this discipline I didn't have so much knowledge. But when I studied nursing I gained the knowledge I needed. When you become familiar with a series of diseases, you know what to do to prevent them. Well, such diseases may exist in my family as well.”* (Participant No. 18) **

The arrival to nursing discipline had caused the students to become curios about their own health. Enthusiasm for more studies and screening tests stimulated them to have periodical tests and identify probable diseases:

*“On the other hand, as I had become a nurse, I liked to study more and know about things. So I worked on my own screening system. For this purpose I went and had myself tested once every 3-4 months.” *(Participant No. 12) **

### 3.5. Needs

The need to have a healthier body in nursing discipline was one of considerable conceptions among the students, which made them observe health-promoting behaviors including nutrition and exercise:

*“Well, I used to eat fast foods so much. But when I started exercise, I stopped eating fast foods for a couple of years. Another reason was our field of study, namely nursing*.” (Participant No. 4) **

Giving priority to health in order to be able to take care of patients better was another need conceived by the participants:

*“The first priority is our own health and the second is other's health. When you are not healthy you won't be able to help others.” *(Participant No. 19) **

Among needed actions in the faculty to promote students' health was to design a system in hospitals to separate work area of physicians from that of nurses and protect the dignity of nurses. Independency of nursing faculties was mentioned as a need:

*“If a system is designed in hospitals to separate work area of physicians from nurses, so that nurses are not ordered by physicians too much, it would be very better. They should respect each other. The main head of the students who go to hospital should be the faculties, not hospital or head nurse.” *(Participant No. 19) **

Furthermore, the participants believed that it is necessary to protect themselves against the risks of the work environment as far as possible. In this matter, how to make body contact with a patient was expressed by students:

*“Considering the job we have, it is mentally hard. We have such challenges as how to contact with patients in work environment, how to protect ourselves against probable risks as far as possible.” *(Participant No. 6) **

### 3.6. Attractiveness of Nursing Discipline (Aesthetics)

One of the things done by students was to compare the status of nursing discipline with other fields such as medicine. In this case, the participants had a feeling of humility and humbleness:

*“Well, when you see in the society that your status and dignity in lower than somebody else, say a student of medicine or a general practitioner, you feel they are more important than you and an unconscious sense of humility appears in you.” *(Participant No. 1) **

The participants compared the problems of their own discipline with students of other fields. This problem was more severe in their internship course:

*“A nine-day internship course which is 1 credit has only one permitted absence. Imagine that someone has cold and needs a two-day absence in the middle of the week. It results in deletion of that credit. These are problems that I think students of other fields don't have.” *(Participant No. 6) **

The participants believed that the subjects they had studied are only about the patient. They felt that their mental and physical needs had been disregarded and that they had been sacrificed for patients and health team personnel:

*“Whatever we study is about patient. There should be some lessons about our own health as well. They should teach us a little about our health and how to comply with health standards. This way we can learn our own mental health not the patient's mental health. We always have to be sacrificed for patients and medical team.” *(Participant No. 6) **

One of the participants who had immigrated to Tehran to study nursing pointed out to conditions of dormitory and compared it with his own home. He expressed that while living in dormitory had enhanced his tolerance, at the same time, it had restricted him to much extent:

*“My problem is being away from my family. Despite it is enhancing my tolerance; it prevents me from being what I like to be.” *(Participant No. 5) **

### 3.7. Coping with Negative Influences in Nursing

Among the ways of coping with negative influences in the field of nursing are to try to establish more friendships, communicate with family, do funny things have entertainment with friends, and be happy in resting times in internship courses. Moreover, to have no mental and emotional problems was among considerable things that they pointed to in connection with coping and preventing the problems:

*“We try to enhance our friendship. For example, we try to have fun in rest times or go out with each other to mentally refresh a little. We also try to contact our family members. I think that is the only solution. This way the students can overcome their mental or emotional problems. If a person is emotionally weak, she gets into trouble.” *(Participant No. 6) **

In connection with negative impact of nursing on physical problems resulting from tiredness and fatigue, the participants mentioned they consume drugs to alleviate pain as one of the methods:

*“When I went home at night after C.C.U internship, I applied salicylate to sleep. I couldn't sleep without that.” *(Participant No. 6) **

The participants who lived in dormitory expressed gradual adaptation to bad conditions of dormitory as a strategy to cope with negative impacts. They hoped to adapt more to dormitory in the next semesters:

*“There was beetle in the room and the food was bad. In the second semester I am starting to adapt. There are still many things I cannot accept. But I hope it gets better until third or fourth semester.” *(Participant No. 13) **

Also, good interperson relation in dormitory was expressed as a strategy of coping with probable problems of living with other students:

*“In dormitory we should protect our rights and behave in such a manner to neither oppress others or to be oppressed. We should respect the rights of others and at the same time we should not let others to order us frequently and consider us a weak person, otherwise we will suffer for the following four years.” *(Participant No. 16) **

Cooperation in compliance with environmental health standards in dormitory was another strategy of coping with environmental issues of dormitory. The students did so as they were aware of the effects of such cooperation:

*“Despite the dormitory have some servants who clean the dormitory every day, the students themselves also clean the dormitory, even the toilets, in cooperation with each other. We all cooperate with each other and try to maintain the dormitory in the best manner.” *(Participant No. 5) **

**Figure 1:**
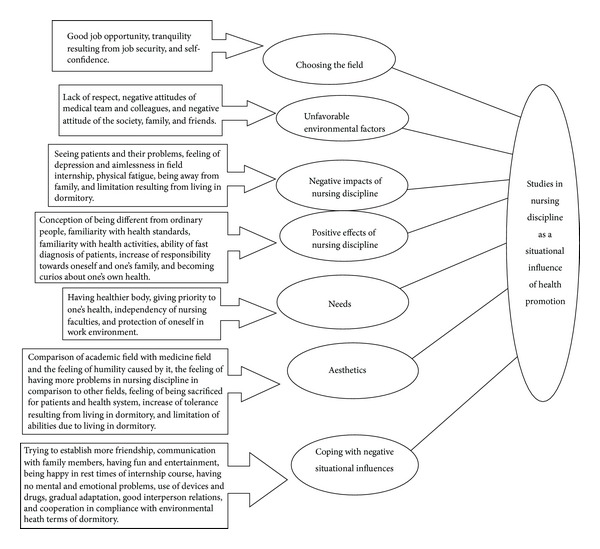
Theme, categories, and subcategories of situational influences perceived in nursing discipline.

The research findings are shown in Figure 1.

## 4. Discussion and Conclusion

The most common influence on the nurses' concentration on health promotion is their personal attitudes [6]. Students have a wide range of perceptions about nursing [17]. In this research, the participants believed that studies in nursing discipline can be a situational factor contributing to their health promotion lifestyle. They expressed that they have used the subjects learnt in nursing discipline to promote their health and their family members' health. In this respect, studies have indicated that nursing comprehensive programs have had positive effects on nursing students, such as considerable improvement of health responsibility and increase of physical, personal, and professional practice use. These changes last for one to seven years [28].

The students enumerated the lack of personal interest, lack of respect for nursing, and negative attitude of the public towards nursing as unfavorable environmental factors. They believed that the biggest damage is inflicted on them by their own colleagues. Negative attitude to nursing and lack of respect for nursing in the society were considered to be connected with field comparison. In this respect, the findings of a research conducted by Kemppainen et al. indicated that cultural aspects of the organization in which nurses' work contributes to their health-promoting activities. This cultural context may be supportive or disappointing [6]. In a study with qualitative approach by content analysis method, it was indicated that the problems of communication with students are one of the important factors contributing to reduction of their interest in nursing discipline. According to the data obtained by analyzing handwritings, all nursing students pointed to cultural-social issues as one of the important factors contributing to reduction of their interest in nursing. This main theme embraced a wide range of factors contributing to reduction of motivation, among which were low dignity and improper conception of the society [29]. The findings have indicated that, in contemporary history, negative image and low dignity of nursing have been among the causes of nurse shortage in Muslim and Arab societies. These negative images of nursing are being continued, and nursing is being considered as a job that requires no skill, has low income, is womanish, and is less important than other healthcare jobs [30]. 

In the present study, nursing discipline has been selected mostly due to its good job opportunity. In other words, the feeling of financial safety was the reason for choosing this field. In this connection, 68 interviews have been made about Saudi Arabian nurses' conception of nursing as a job. The findings indicated that despite negative conceptions of gender in nursing, the conception of good job opportunity of this field was increasing [30].

In this research, nursing's negative impacts on the students' health were mostly mental problems and feeling of tiredness, in such a way that intensiveness of the courses had made them unable to have good nutrition, meet their physical and mental needs and have sufficient entertainment and rest. In this matter, the studies performed on nurses have indicated several themes that evaluate health promotion in nurses as weak. Among the things expressed are insufficient sleep, smoking and improper nutrition in surgery room nurses, the need to improve health activities in special care units nurses, improper nutrition and exercise in American-African nurses, and the need to concentrate on nutrition, exercise, and stress management [28].

Among the positive effects of studies in nursing discipline enumerated by the participants were having more knowledge than the public community, ability of early diagnosis of their diseases, awareness about health standards and better care of oneself. Studies have indicated that passing nursing discipline subjects which concentrate on health promotion has undeniable effects on the increase of students' level of knowledge. A research made by Hong et al. showed that there is a positive meaningful relation between health promoting lifestyle and courses and subjects of health-promotion passed in nursing discipline [31].

The students expressed the need to have a healthier body in nursing discipline in order to take better care of patients, which resulted in improvement of health-promoting behaviors such as nutrition and exercise. In order to advocate healthy life style and to be an effective model, nurses are expected to pay more attention to their own health [1].

The participants also expressed that nursing faculties should be independent. According to the previous studies, nursing can be studied as a mummified job in which old and stereotyped values have highly remained. The main motivation to become a nurse is the desire for independency, flexibility, and altruism. In order to enhance nursing profession, professors and clinical supervisors should consider nursing students' attitudes as an important point of education and develop them in different environments [32].

Among the issues expressed by the participants was comparison of nursing's status with other fields of study. They compared the problems of their field with the problems faced by students of other fields and estimated their health to be in a lower level. Furthermore, the students who lived in dormitory had conceived the dormitory's environment as unpleasant. Two studies have indicated that stressful environments result in unhealthy solutions such as smoking and improper nutrition, which in turn causes harmful behaviors having negative impact on patients' health and reduction of efficiency of the nurses as health promoters [33]. According to findings of a research on the attitudes of others towards nurses' health-promoting behaviors, the participants felt that their efforts had been understood positively by patients. However, some of the participants believed that their work environment is unsupportive [34]. 

In the present research, the participants believed that the subjects they have learnt are merely about patients. They felt that they had been sacrificed for patients and medical team. In this respect, McElligott et al. believed that nurses often use holistic approaches to consult patients on health promotion. However, studies have denoted that there are weak health-promoting behaviors in the nurses themselves [28].

Among the strategies of coping with negative influences in nursing discipline are establishment of more friendships, communication with family members, having fun and entertainment with friends and family, and being happy in rest times of internship courses. In this respect, the results of studies have indicated that excessive occupational needs, fear of errors, unsafe work environment and weak communications are among health promotion obstacles for nurses. The inclusion of welfare programs in work place will have a positive effect on health promotion and results in improvement of health, enhancement of creativity, satisfaction, and reduction of health care costs [28].

## 5. Limitations and Recommendations

Since the present research studied a limited number of students, its generalization to other conditions and situations must be performed with caution. It is recommended that some research be made on the perceived situations of nursing in broader dimensions and in other educational levels of nursing discipline.
